# An Effective Conversion of Visemes to Words for High-Performance Automatic Lipreading

**DOI:** 10.3390/s21237890

**Published:** 2021-11-26

**Authors:** Souheil Fenghour, Daqing Chen, Kun Guo, Bo Li, Perry Xiao

**Affiliations:** 1 School of Engineering, London South Bank University, London SE1 0AA, UK; chend@lsbu.ac.uk (D.C.); perry.xiao@lsbu.ac.uk (P.X.); 2 Xi’an VANXUM Electronics Technology Co., Ltd., Xi’an 710129, China; guokun84@126.com; 3 School of Electronics and Information, Northwestern Polytechnical University, Xi’an 710129, China; libo803@nwpu.edu.cn

**Keywords:** deep learning, lip reading, neural networks, speech recognition, robustness, augmentation, visemes, Gated Recurrent Unit, recurrent neural networks

## Abstract

As an alternative approach, viseme-based lipreading systems have demonstrated promising performance results in decoding videos of people uttering entire sentences. However, the overall performance of such systems has been significantly affected by the efficiency of the conversion of visemes to words during the lipreading process. As shown in the literature, the issue has become a bottleneck of such systems where the system’s performance can decrease dramatically from a high classification accuracy of visemes (e.g., over 90%) to a comparatively very low classification accuracy of words (e.g., only just over 60%). The underlying cause of this phenomenon is that roughly half of the words in the English language are homophemes, i.e., a set of visemes can map to multiple words, e.g., “time” and “some”. In this paper, aiming to tackle this issue, a deep learning network model with an Attention based Gated Recurrent Unit is proposed for efficient viseme-to-word conversion and compared against three other approaches. The proposed approach features strong robustness, high efficiency, and short execution time. The approach has been verified with analysis and practical experiments of predicting sentences from benchmark LRS2 and LRS3 datasets. The main contributions of the paper are as follows: (1) A model is developed, which is effective in converting visemes to words, discriminating between homopheme words, and is robust to incorrectly classified visemes; (2) the model proposed uses a few parameters and, therefore, little overhead and time are required to train and execute; and (3) an improved performance in predicting spoken sentences from the LRS2 dataset with an attained word accuracy rate of 79.6%—an improvement of 15.0% compared with the state-of-the-art approaches.

## 1. Introduction

The automation of lipreading has attracted a significant amount of research attention in the last several years. A variety of different approaches have been utilised for classification, with deep learning-based approaches being particularly popular for lipreading individuals uttering words and sentences.

Lip movements can be decoded by using a variety of forms, including visemes, phonemes, characters, and words. Accordingly, each of these forms can provide a different classification schema for designing automated lipreading systems. Such systems vary in their capabilities, ranging from recognising isolated speech segments in the form of individual words or characters to decoding entire sentences covering a wide range of vocabulary. In some cases, the lexicons can consist of a vocabulary with thousands of different possible words.

Visemes are the fundamental building blocks of visual speech, and visual speech recognition is a task of significant importance when audio is unavailable or when there is noise, which makes audio speech recognition difficult or even impossible. Using visemes as classes for automated lip reading has the following advantages:

1. Fewer classes are needed in comparison to the use of ASCII characters, and words which can reduce computational bottleneck;
2. Pre-trained lexicons are not required. Hence, in theory, a viseme-based lipreading system can be used to classify words that may have not been presented in the training phase. This is because visemes can be classified as images where deciphered visemes can then be matched to all the possible words spoken;
3. They can be applied to different languages because many different languages share the same visemes.

However, the overall performance of a viseme-based lipreading sentence system has been significantly affected by the efficiency of the conversion from visemes to words. In some cases, a high accuracy for visemes classification can be achieved, for instance, in the work by Fenghour et al. [1]: more than 95.4% accuracy of viseme classification was achieved; however, the overall accuracy of word classification dropped down to 64.6%. The underlying cause of this phenomenon is the existential problem whereby one set of visemes can map to multiple different sounds or phonemes resulting in a one-to-many relationship between sets of visemes and words. As such, it becomes critical to design efficient strategies for viseme-to-word conversion in order to develop practically useful viseme-based lipreading systems.

In this paper, a viseme-to-word converter is proposed for effectively distinguishing between words that share identical visemes, and its performance is compared with three other approaches. When compared with other approaches [2,3,4,5,6], the proposed approach is more robust to the possibility of misclassified visemes in the input, and its robustness is demonstrated by adding perturbations to the input visemes and comparing the outputs to the ground truth. Moreover, the converter is integrated within a deep learning network-based architecture for lip reading sentences and is trained on the LRS2 dataset; it attained an improved performance of over 15% on other lipreading systems evaluated on the same corpus such as Ma et al. [7] (a word accuracy of 62.1%) and Fenghour et al. [1].

The four models being compared include an Attention-based GRU (Gated Recurrent Unit Networks), Perplexity-Iterator, Feed-Forward Neural Network, and Hidden Markov Model. The rationale behind the comparison of these models is to compare conversion approaches from three different categories of conversion implementation, discussed in more detail in Section 2. One of the approaches uses a statistical language model with a fixed-context window while another of the approaches uses a feed-forward neural network with a fixed-context window. The other two approaches use neural language models; however, one of these approaches is known to perform poorly when there are incorrectly classified visemes as inputs because the predicted output is vulnerable to error propagation.

The best proposed approach has been experimentally verified. It was shown to be more effective at discriminating between words sharing visemes that are either semantically or syntactically different. This is because unlike other approaches that only use context from a fixed-window, the proposed approach uses unlimited context to detect semantic and syntactic information needed for disambiguation. The proposed approach is also shown to be somewhat robust to incorrectly classified visemes due to its ability to model both long and short term dependencies. The novelty of this paper is not any model being proposed but the application of a model in the domain to solve a new problem in converting visemes to words and exploiting sufficient context to overcome the one-to-many mapping problem.

The rest of this paper is organised as follows: First in Section 2, a literature review is provided, describing the general approaches used for viseme-to-word conversion, their limitations and advantages of the proposed approach, and an explanation of how the conversion model for the proposed viseme-to-word converter addresses the limitations of existing converters. Then, in Section 3, all the distinct components that make up the viseme-to-word conversion process are described, including the following: the principle of perplexity analysis, the neural network used for performing viseme-to-word conversion, the data augmentation techniques used for modelling the converter’s robustness to noise, and the accuracy metrics used to evaluate the performance of the word detector. In Section 4, the performance of the proposed word converter is discussed and compared with other approaches, followed by concluding remarks provided in Section 5 along with suggestions for further research.

## 2. Literature Review

Viseme-to-word conversion is related to a language model and the various methods of the implementing a language model. Conversion methodologies used to predict words from visemes can be grouped into two categories: statistical conversion models and neural conversion models. This section provides the essential fundamentals of a language model and the different methods of implementing of a language model in order to analyse how effective they are when applied in a viseme-to-word conversion model.

### 2.1. Implementation of a Language Model

A language model is a probability distribution over sequences of words, and it can be measured on the basis of the entropy of its output from the field of information theory [8]. A language model provides context to distinguish between words and phrases that look similar when spoken. For example, the phrases “recognize speech” and “wreck a nice beach” both appear similar when uttered and consist of identical lip movements. The context of a word in a language model can be deduced by its surrounding words, and according to the linguist J. R. Firth, “you shall know a word by the company it keeps” [9].

The language model will predict the most likely set of words that was spoken given the spoken visemes, and the two methods for implementing a language model include statistical language models and neural models. Statistical language models predict words based on the preceding words in the sequence according the Markov assumption, whereas neural language models use deep neural networks.

Algorithms such as Weighted Finite State Transducers (WFSTs) [10] and HMMs [11] are some examples of statistical conversion models as they implement language models based on Markov chains or N-grams, which assume that each word in a sentence depends only on its previous N−1 predecessors.

Statistical models based on N-grams are limited in comparison to neural models because they are a sparse representation of language in which model sentences based on the probability of words in combination and would naturally provide a zero probability to combinations of words that have not previously appeared [12]. Furthermore, N-grams fail to accurately predict semantic and syntactic details of sentences [12]. However, one fundamental problem with N-grams is that they need a large value of *N* to produce an accurate language model, which requires a lot of computational overhead.

An N-gram model predicts sequences of words according to the Markov process where the probability of the next word in a sequence is predicted based on the previous (N−1) words. Equation (1) provides the ideal chain rule of probability *P* to apply to any language model with a sequence of *K* words. However, as *K* increases, the computation becomes impossible, so statistical language models use the Markov assumption given in Equation (2).

(1) $$P\left(w_{1},w_{2},\ldots ,w_{K}\right)=\prod_{i}P\left(w_{i}|w_{1},w_{2},\ldots ,w_{i-1}\right)$$

(2) $$P\left(w_{1},w_{2},\ldots ,w_{K}\right)=\prod_{i}P\left(w_{i}|w_{i-N+1},\ldots ,w_{i-1}\right)$$

N-grams are an approximation of the Markov assumption, and the problem with N-grams is that the context is only limited to the preceding N−1 words (Equation (3)). Moreover, although one exploits more contextual information by increasing the value of *n*, this comes at the cost of increasing the computation of the model [13]. Bigrams language models will only be able to predict words based on the previous word in a sentence (Equation (4)), which in practice is insufficient for disambiguating words sharing identical visemes.

(3) $$P\left(w_{i}|w_{i-N+1},\ldots ,w_{i-1}\right)=\frac{count(w_{i-N+1},\ldots ,w_{i-1},w_{i})}{count(w_{i-N+1},\ldots ,w_{i-1})}$$

(4) $$P\left(w_{i}|w_{i-1}\right)=\frac{count(w_{i},w_{i-1})}{count(w_{i-1})}$$

One major difference between statistical models and neural models is that whilst the former treats each word as a fixed representation such as a one-hot-vector [13], neural models use the concept of distributed representations whereby words are treated as continuous vectors, each with a discrete number of features(each feature represents a semantic dimension in feature space). This means that words that are semantically similar are closer together in vector space. Neural models are a dense representation of language that avoid what is known as the curse of dimensionality [13].

Feed-forward neural networks are an example of a neural conversion model. They have advantages over statistical conversion models modelling n-grams that use HMMs or WFSTs in that they are not limited by data sparsity or the inability to learn semantic and syntactic information, which means that they can even model unseen combinations of words not observed in training. Modelling unseen word combinations is necessary for ensuring that the viseme-to-word converter is not limited to predicting combinations of words only observed in training for any given combination of words.

The output of a feed-forward network at a certain timestep will always be conditioned on a window of the previous N−1 outputs, which a softmax layer is applied to. As observed in Equation (5), the fully connected layer $a_{k}$ (for class *k* corresponding to one of *N* classes) uses only hidden state from the previous n−1 steps. Increasing the window size requires more weight parameters, which increases the complexity of the model [13].

(5) $$P\left(w_{t}=k|w_{t-n+1},\ldots ,w_{t-1}\right)=\frac{e^{a_{k}}}{\sum_{i=1}^{N}e^{a_{i}}}$$

However, similarly to N-grams, feed-forward networks still suffer from the fundamental problem in that they use fixed-size windows to give context whereby the output of a timestep is only conditioned on a limited number of previous timesteps. They are not always able to utilise all the context necessary in exploiting semantic or syntactic information needed to distinguish between words that share identical visemes.

Recurrent Neural Networks (RNNs) on the other hand are capable of conditioning the output of a model on all the previous words in a sentence. Equation (6) provides the expression for the hidden state $h_{t}$, which is dependent on the current input $x_{t}$ at time *t*, and hidden state from the previous step $h_{t-1}$, which will in turn be dependent on the output of previous timesteps. The hidden state will, therefore, always be dependent on the hidden state from all previous timesteps. The output of a particular timestep $y_{t}$ is given in Equation (7). Unlike the feed-forward network, RNNs are not constrained by sequence length, and longer sentences have no effect on the weight parameters [14].

(6) $$h_{t}=H\left(W_{xh}x_{t}+W_{hh}h_{t-1}+b_{h}\right)$$

(7) $$y_{t}=W_{hy}h_{t}+b_{y}$$

**Figure 1:**
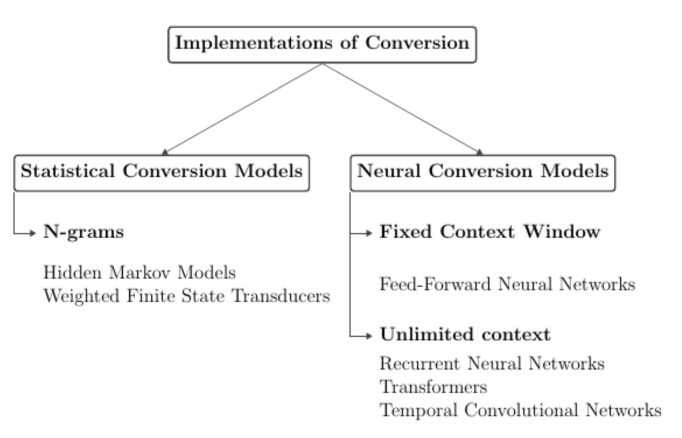
Taxonomy of viseme-to-word conversion models.

Statistical models predict words according to ratios of counts for sequences of words within a window of *n* words according to Equation (8). Neural models with a fixed context predict words according to the relationship of feature vectors within a fixed window of *n* words according to Equation (9). Neural models with unlimited context predict words according to the relationship of feature vectors for all previous words (Equation (10)). Figure 1 shows the taxonomy of the different viseme-to-word conversion models. They can be decomposed into statistical models, neural models with fixed context, and neural models with unlimited context.  

(8) $$P\left(w_{t}|w_{1},\ldots ,w_{t-1}\right)=\frac{count(w_{t-N+1},\ldots ,w_{t-1},w_{t})}{count(w_{t-N+1},\ldots ,w_{t-1})}$$

(9) $$P\left(w_{t}|w_{1},\ldots ,w_{t-1}\right)=f\left(w_{t}|w_{t-n+1},\ldots ,w_{t-1}\right)$$

(10) $$P\left(w_{t}|w_{1},\ldots ,w_{t-1}\right)=f\left(w_{t}|w_{1},\ldots ,w_{t-1}\right)$$

Most RNNs used for language modelling take the form of either LSTMs (Long Short Term Memory Networks) or GRUs because traditional vanilla RNNS are susceptible to the problem of vanishing or exploding gradients for very long sequences. LSTMs and GRUs have memory cells to alleviate this problem. A GRU [15] consists of memory cells with weights *W* and a function *H* applied to the input according to Equation (5). Each cell at a timestep *t* will have an input gate *x*, update gate u and reset gate *r*. All parameters are updated according to Equations (11)–(14).

(11) $$h_{t}=u⊗{\tilde{h}}_{t}+\left(1-u\right)⊗h_{t-1}$$

(12) $$u_{t}=\sigma \left(W_{xu}x_{t}+W_{hu}h_{t-1}+b_{u}\right)$$

(13) $${\tilde{h}}_{t}=\tanh\left(W_{xh}x_{t}+W_{hh}\left(r⊗h_{t-1}\right)+b_{h}\right)$$

(14) $$r_{t}=\sigma \left(W_{xr}x_{t}+W_{hr}h_{t-1}+b_{r}\right)$$

GRUs are able to select whether a unit for a timestep should have short or long term dependency. Reset gates help to capture short-term dependencies while update gates capture long terms dependencies, and this helps GRUs in ignoring parts of sequences when needed. The reset gate *r* and update gate *u* can be switched on and off by containing values close to one and zero, respectively, and Equations (15)–(17) have been derived, indicating how a GRU behaves when the reset and update gate variables approach asymptotic limits.

A GRU behaves similarly to a vanilla RNN when both gates are switched on, as indicated by Equation (15). When the update gate is switched off, the hidden state gives more attention to the previous hidden states (Equation (16)), while setting off the reset gate would cause the GRU to give more attention to the current input at that timestep (Equation (17)). With this in mind, a GRU-based language model is better at modelling shorter length dependencies within a sentence for values of *r* close to zero, which make it less susceptible to the possibility of compound errors.

(15) $$\lim_{\left(u,r)\to (1,1\right)}h_{t}=\left(W_{xh}x_{t}+W_{hh}h_{t-1}+b_{h}\right)$$

(16) $$\lim_{\left(u,r)\to (0,1\right)}h_{t}=h_{t-1}$$

(17) $$\lim_{\left(u,r)\to (1,0\right)}h_{t}=\left(W_{xh}x_{t}+b_{h}\right)$$

### 2.2. Comparison of Viseme-to-Word Conversion Models

Table 1 provides a summary of some of the approaches to two-stage visual speech recognition that use visemes as the intermediate class. TCD-TIMIT [16], LiLiR [17], RM-3000 [18], and LRS2 [19] are examples of sentence-based audio-visual datasets that were used for training and validation which contain videos of different people speaking a variety of sentences.

In one study by Fenghour et al. [20], a Long-Short Term Memory Network (LSTM) was used that takes visemes as an input and predicts the words that were spoken by individuals from a limited dataset with some satisfactory results. This configuration presupposes that the identities of individual visemes are already known; thus, its robustness to misclassified visemes has not been verified. Moreover, the sentences that are predicted are often not grammatically sound in terms of syntax, and many sentences predicted incorrectly have large grammatical perplexity.

Lan and Harvey [5] classified words from decoded visemes using HMMs with a bigram language model to predict words once spoken visemes had been classified from videos of spoken sentences from the LiLiR corpus. Visemes were classified with an accuracy of 45.67% while the word accuracy achieved was 14.08%.

Thangthai et al. [3] decoded visual speech in the form of both visemes and phonemes for four different first-stage classification methods whilst using a WFST for the second-stage conversion when predicting spoken words. Every one of the four systems performed viseme classification with greater accuracy than phoneme classification. However, a greater accuracy was observed at the second stage in the word conversion process because the efficiency in performing phoneme-to-word conversion was higher than that of the viseme-to-word conversion. The main reason for better results being achieved when using phonemes instead of visemes is that there will be always be more ambiguity with the use of visemes as there are significantly more mapping options available [3].

It may seem inherent that the intermediate units to be modelled should be visemes when there is no audio available. However, the availability of context and good accuracy being attained at the first stage, would make the use of phonemes more preferable for the prediction of sentences than visemes. The increased ambiguity that one has to overcome with the use of visemes as opposed to phonemes is due to there being far more words in the English language that share visemes than phonemes [2,3,21,22,23], meaning that there are significantly fewer mapping options to be considered when performing conversion for word prediction.

It is for this reason that Howell et al. [2] preferred to use phonemes as the intermediate class. They acknowledge that even with both perfect feature extraction and a perfect performance for first stage classification, second stage conversion will always be limited because an HMM or WFST based conversion model will fail to predict semantic details when distinguishing between semantically different words such as “Hepburn”/“Campbell”, “barge”/“march”, and “six”/“since” or syntactic details when there is confusion of plural and singular versions of a word that ends with a viseme corresponding to same for the letter “s”, e.g., “threat”/“threats” [2].

Almajai [6] experimented with three different methods of classifying visemes including Linear Discriminant Analysis(LDA) with an HMM, LDA with Maximum Likelihood Linear Transform(MLLT), and an LDA/MLLT/Speaker Adaptive Training(SAT) hybrid; but it was the LDA+MLLT+SAT classifier that recorded the best result for viseme classification. They then used two different algorithms, an HMM and a feed-forward neural network, to perform word conversion, and the feed-forward network was the better performing network of the two, recording an accuracy of 47.75% compared with the feed-forward network achieving 37.71%.

The lip reading system proposed by Fenghour et al. [1] used a viseme-to-word converter to match clusters of visemes to word combinations by iteratively combining words and calculating perplexity scores. A Generative Pre-Training (GPT)-based transformer [24] is used to calculate perplexity scores of word combinations in order to determine the most likely combination of words given the clusters of visemes that are inputted. Perplexity is a measure of grammatical correctness; thus, it is expected that the most likely combination of words to have been uttered given a set of visemes is the combination with the lowest perplexity score.

The model used by Fenghour et al. [1] matches clusters of visemes to words in a lexicon mapping and is contingent on visemes being classified correctly. Visemes being misclassified in one cluster will not only cause error in word matching for that one word but will in turn cause compound errors in the combination process during the iterations due to the conditional dependence of word combinations. This means that one word being misclassified can cause other words to also be misclassified as well.

Other important studies in the field that have utilised visemes as part of a two-stage conversion process include Sterpu and Harte who used a Discrete Cosine Transformation with an HMM to classify visemes with an HMM for word conversion [25]. Peymanfard et al. used a neural network architecture consisting of a Convolutional Neural Network(CNN) frontend with an attention transformer backend to classify visemes and then a attention-transformer to predict words [26]. The full results of the viseme classification for both these works has not been disclosed.

Many of the conversion models listed in Table 1 lack the discriminative power to be able to learn semantic and syntactic information needed to distinguish between words that share identical visemes [2]. This is because of the lack of context available due to their fixed size context windows, and to increase the size of the context window only increases the computational complexity of the model. The conversion model proposed here uses a GRU network that can exploit context from an unlimited number of timesteps regardless of the length of sentence, which itself would not affect model complexity.

In addition to being effective at exploiting unlimited previous context to discriminate between words sharing identical visemes, the proposed conversion model is also robust to misclassified visemes because it can capture both long and short term dependencies unlike the conversion model used by Fenghour [1]. It is, therefore, less prone to cascading errors.

Another limitation of the word converter used in [1] is its inefficiency. The architectural model proposed in this paper uses significantly less parameters and takes significantly less time in executing the prediction of a spoken sentence for one viseme sequence.

The word converter proposed in this paper is trained on a large dataset with a wider range of vocabulary than the converter in Fenghour et al.’s work [20], which was only trained on words and sentence contained in the much smaller and limited TIMIT corpus [27]. A curriculum learning strategy such as that of Chung et al. [20] was also used in the training phase to ensure that the network can better model natural language by predicting shorter N-grams. The proposed approach falls into the category of neural conversion models.

For a viseme-to-word converter to be accurate, it has to be effective in classifying words from visemes that have been classified correctly but must also be robust to the possibility of visemes that have not been classified correctly. This section is devoted to presenting theoretical justifications for why the proposed approach is more effective in addressing these scenarios than other conversion methods.

It is apparent that the majority of viseme-to-word converters that used either HMMs, WFSTs, or even Feed-forward networks were ineffective with a low conversion performance. The reason for this is that such models are unable to use enough context to disambiguate between commonly confused words that share visemes. In Section 2.3, an explanation is given as to why the Attention based GRU model [28] proposed is more effective in discriminating between words corresponding to identical visemes, and it is because they are able to use more contextual information in extracting lexical rules to learn the syntactic and semantic differences between words.

The GPT-based iterator used in [1] that predicts words using perplexity calculations has a very high conversion performance for correctly classified visemes; however, it is nonetheless is highly susceptible to the presence of incorrectly classified visemes. Incorrectly classified visemes result in wrong predictions for the individual word which in turn causes error propagation in the predicted word sequence of the outputted sentences. The GRU model proposed here is supposed to be susceptible to the impact of incorrectly classified visemes due its ability to model both short and long term dependencies.

### 2.3. Syntactic and Semantic Disambiguation

Given the visemes decoded, a language model is required in order to determine the most probable combination of words to have been uttered and it has to be robust to the possibility of visemes being misclassified. According to Equation (18), for a set of given visemes *V*, a language model will predict the most likely set of words $W^{*}$ that have been uttered for different combinations of words *W* [29]. Table 2 provides an example of a set of visemes and the words that are most likely to correspond those visemes.

(18) $$W^{*}=\arg\max_{W}P\left(V|W\right)P\left(W\right)$$

Classifiers with language models that have been previously used for viseme-to-word conversion such as N-grams have been ineffective due to the algorithms’ inability to discriminate between words that share visemes but are different either syntactically or semantically. An example of syntactically distinct words sharing identical visemes would be the case of plural and singular versions of a word that end with a viseme corresponding to a consonant with same viseme as that for the letter “s”. Examples of semantically distinct words sharing identical visemes that are confused are those words that have an identical likelihood of being preceded by a common word in a bigram [2].

If the context window is long enough, it can capture subject-verb agreement, which is a grammar rule that can be used to determine if a noun is singular or plural and, thus, address the problem of syntactic disambiguation. The subject-verb agreement is a situation whereby the status of a noun subject being singular or plural can be determined by the form of the verb. If one takes the sentence “the keys to the cabinet are on the table” as an example, the word “keys” is in agreement with the word “are” and if enough context is captured, the correct syntactic form of the noun can be determined [30].

To maximise the probability of distinguishing between words that are syntactically different, one would need to utilise context either side of the subject noun meaning that both left and right context [31] are required and unidirectional RNNs will only be able to exploit left context. Bidirectional RNNs can exploit left and right context; however, a bidirectional network uses twice the number of parameters and more computational overhead to train and evaluate the model.

Intuitively, one can conclude that having access to greater context provides language models more discriminative power and would help to differentiate between words that are semantically different. A noun can be disambiguated by using relationship analysis. Noun phrases will follow different patterns that are characterised between the different types of words that noun phrases contain. The phrase categories can be narrowed to adjective-noun phrases, verb-adjective-noun phrases, and subject-verb-object phrases [32]. The identity of a noun can be determined by the adjective describing it or the verb action it is performing, and the more context there is available for a language model, the greater the probability of it being predicted.

## 3. Methodology

Given a sequence of visemes, the objective is to predict the possible sentence spoken by someone given that only the lip movements or visemes of the person speaking are known and given that there is the possibility of an error or misclassification in the input viseme sequence. In this section, an overall framework is proposed for the viseme-to-word conversion with a component for testing its robustness to misclassified viseme sequences. The entire process consists of two main components: a viseme-to-word conversion model for performing the viseme-to-word conversion and a noisifying component for performing data augmentation to vary the errancy of the input visemes. The performance of the system is evaluated by comparing sentences predicted by the viseme-to-word converter to the ground truth of the actual sentences and then measuring the edit distance [33] (which is the minimum number of character-level operations required to correct the actual sentence to the ground truth).

One aim of this study is to model and improve upon the performance of viseme-to-word conversion reported in another study [1]—particularly with attention to misclassified visemes because the word detector used relies on visemes being decoded with 100% precision. In addition to modelling the robustness of the viseme-to-word converter, the conversion performance attained is also sufficient for solving the problem of why visemes are not widely used as a classification schema in lip reading, which is down to the performance of viseme-to-word conversion being inadequate because of the failure to pick up on semantic and syntactic information needed to distinguish between words that share identical visemes (as discussed in Section 2.3). Four models have been utilised to convert visemes to words directly, and these include the following:

- An Attention based sequence model using GRUs;
- A GPT-based iterator that uses perplexity scores;
- A Feed-Forward Neural Network;
- A Hidden Markov Model.

**Figure 2:**
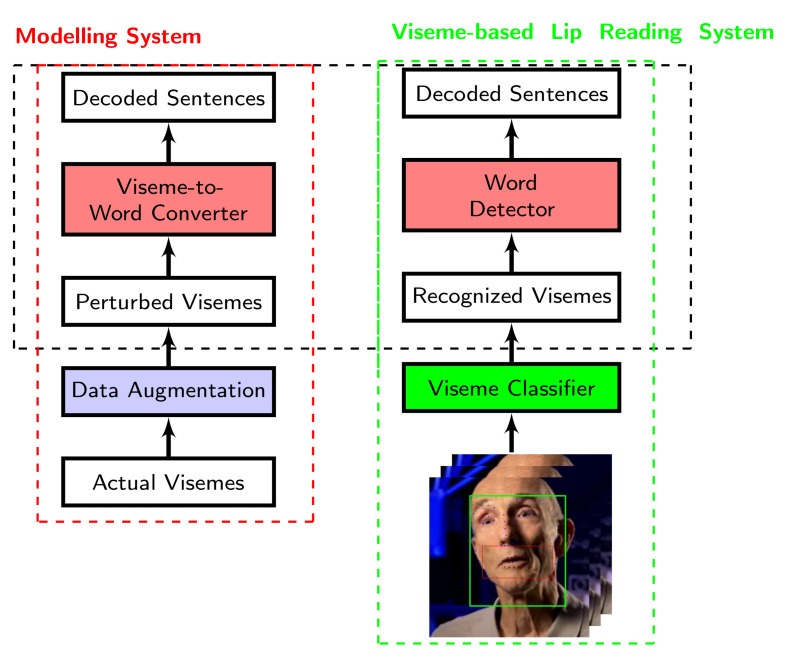
Modelling of viseme-to-word conversion.

Figure 2 outlines the framework of a lip reading system that uses solely visual cues. This framework comprises a viseme classifier followed by a viseme-to-word converter where an image-based classification system takes image frames of a person’s moving lips to classify visemes. Once the visemes have been recognised, a word detector uses the output of the viseme classifier as the inputs in order to determine which words were spoken. For the conversion of visemes to words, a variety of sequence modelling networks could be used to determine the most likely set of words to have been uttered given the visemes that had been decoded.

Some sequence-modelling approaches are prone to the possibility that one incorrectly decoded word causes the other words in the rest of the outputted sequence to also be predicted incorrectly. This is why, for the purpose of evaluating the robustness of a viseme-to-word converter, data augmentation techniques are used to add noise to viseme sequence using techniques that include deletion, insertion, substitution, and swapping so as to model the performance of the viseme-to-word converter under varying levels of noise. The augmentation or noisification techniques are described in more detail in Section 3.4. However, robustness performance results have only been reported for the GRU model and GPT-based iterator because the priority of a viseme-to-word converter is its efficiency.

Overall, there are three instances that the viseme-to-word detection’s performance is being reported. For all three of these instances, their respective performances will be compared with the perplexity-based viseme-to-word converter discussed in Section 3:

1. Visemes with 100% accuracy where the identity of spoken visemes are known;
2. The outputs of the viseme classifier reported in [1];
3. Perturbed visemes with added noise whereby the errancy is varied.

### 3.1. Data

The dataset used in this research is the LRS2 dataset [19]. It consists of approximately 46,000 videos covering over 2 million word instances, a vocabulary range of over 40,000 words, and sentences of up to 100 ASCII characters from BBC videos. This paper is all about modelling how robust the viseme-to-word converter is to noise and misclassifications; thus, the details about the videos will not be discussed here. Additionally, videos from the LRS3-TED dataset [34] have also been used for Scenarios 1 and 3 pertaining to visemes with 100% accuracy where the identity of spoken visemes are known and where the errancy of visemes is varied. This dataset is more challenging because the sentences are on average longer in length and it consists of a vocabulary covering over 50,000 possible words.

Lip reading datasets such as LRS2 consist of labels in the form of subtitles. These subtitles are strings of words that need to be converted to sequences of visemes in order to provide labels for the viseme classifier. The conversion is performed in two stages: First, they are mapped to phonemes using the Carnegie Mellon Pronouncing Dictionary [35], and then the phonemes are mapped to visemes according to Lee and Yook’s approach [36]. The GRU-based viseme-to-word converter uses 17 classes or input tokens in total; these include the 13 visemes, a space character, start of sentence (SoS), end of sentence (EoS), and a character for padding. All the defined classes are listed in Table 3. All viseme sequences are padded to 28 characters, which is the length of the longest viseme sequence.

There is no official standard convention for defining precise visemes, and even the precise total number of visemes and different approaches to viseme classification have used varying numbers of visemes as part of their conventions with different phoneme-to-viseme mappings [8,36,37,38,39,40]. All the different conventions consist of consonant visemes, vowel visemes, and one silent viseme, but Lee and Yook’s [36] mapping convention appears to be the most favoured for speech classification, and it is the one that has been utilised for this paper. It is accepted, however, that there are multiple phonemes that are visually identical on any given speaker [41,42].

For the classifier, an output token will be assigned for every single word that is contained within sentences that make up labels for the training data, and there will be four additional tokens: “<start>,” “<end>,” “<pad>,” and “<unk>.” The “<start>” token becomes appended to the start of every sentence, with the “<end>” token being appended to the end of every sentence, while “<unk>” is a token for modelling any words that do not appear in the training phase.

### 3.2. Viseme Classifier

The Viseme Classifier used is identical to that used in [1], and it relies on the same Preprocessing and Visual Frontend. Videos consist of images with red, green, and blue pixel values and a resolution of 160 pixels by 160 pixels, plus a frame rate of 25 frames/s. Identical pre-processing is used whereby videos proceed through the same stages of sampling, facial landmark extraction, grayscale conversion, and cropping around the boundary of the facial landmarks. Image data augmentation is then applied in the form of horizontal flipping, random frame removal, and pixel shifting, which is then followed by z-score normalisation. This results in reduced image dimensions of 112×112×T dimensions (where T corresponds to the number of image frames).

The viseme classifier itself follows the transformer [43] architecture with an encoder-decoder structure using multi-head attention layers used as building blocks albeit with modifications to the decoder topology. The encoder used is a stack of self-attention layers and the decoder consists of three fully connected layer blocks. The viseme classifier takes pre-processed images of lips as input to predict sequences of visemes using the 17 classes referred to in Section 3.1. The network was trained on all 45,839 training samples of the LRS2 dataset with 1243 samples used for validation.

### 3.3. Viseme-to-Word Converters

In order to demonstrate the effectiveness of neural language models with unlimited context, the performance of an Attention-based GRU has been compared with two other conversion models, each of which are representative of viseme-to-word conversion models from the two categories listed in Figure 1 of Section 2.2, and these include a bigram Hidden Markov Model (to represent statistical language models) and a Feed-Forward Model (to represent neural language models with a fixed window). A third model in the form of a GPT-based iterator has also included for comparison in performance to demonstrate the Attention-based GRU’s robustness to incorrectly classified visemes.

#### 3.3.1. Hidden Markov Model

The Hidden Markov Model(HMM) is similar to that used by Vogel et al. [44], which was used for a statistical machine translation task. Visemes can be modelled as individual visemes or as clusters where groups of visemes make up a word. Unlike the approaches of works reported in [1,20], this approach classifies words based on sequences of individual visemes. The HMM uses a bigram language model to predict words given the inputted visemes corresponding to one of seventeen tokens, and the language model is accompanied with Laplace smoothing and a Katz backoff. The bigrams used to train the model are all extrapolated from one of either the LRS2 and LRS3 training sets.

#### 3.3.2. Feed-Forward Neural Network

The feed-forward neural network used implements a language model similar to that of Bengio et al. [13] and uses a context window of the same size as HMM model. The network consists of a dense layers with 1024 nodes plus a softmax layer with classes corresponding to all possible tokens contained within one of the LRS2 and LRS3 datasets. The network is trained using the cross-entropy loss function with the training set split into batches of 64 samples each. During the training phase, the Adam optimiser [45] is implemented with default parameters ($\beta_{1}=0.9$, $\beta_{2}=0.999$, and $\epsilon =10^{-8}$) and initial learning rate $10^{-3}$. A curriculum learning strategy is used to train the network, similar to the training performed for Chung et al.’s model [20]. For the first iteration, sentences are clipped to one word followed by two words in the next iteration, then three words, then four, and finally the full length of sentences. The rationale behind this is to better learn the grammatical structure of word combinations found in natural language by being able to learn N-grams of variable lengths.

#### 3.3.3. GPT-Based Iterator

For a spoken sequence of visemes $V=(v_{1},v_{2},\ldots ,v_{N})$ where $v_{i}$ corresponds to every *i*th viseme, $W=(w_{1},w_{2},\ldots ,w_{N})$ represents any given combination of words that map to those visemes, where $w_{i}$ corresponds to every *i*th word within the string of words. Given that visemes have a one-to-many mapping relationship with phonemes, which results in a situation of a cluster of visemes that map to several different words, it is expected that the combination of words that are most likely to have been uttered would be the combination that is most grammatically correct and, thus, the combination with the greatest likelihood of occurrence. The string of words $\overset{ˇ}{W}$ that is expected to have been uttered for a set of visemes would be the combination that has the greatest likelihood. A sequence of visemes *V* can map to any combination of words $W_{C}$ for a combination *C* that falls within the overall set of combinations $C^{*}$. The solution to predicting the sentence spoken is the combination of words given the recognised visemes, which has the greatest probability as expressed in Equation (19).

(19) $$\overset{ˇ}{W}=\arg\max_{C\epsilon C^{*}}P\left(W_{C}|V\right)$$

Perplexity is a measure of the quality of a language model, because a good language model will generate sequences of words with a larger probability of occurrence resulting in smaller perplexity. The Perplexity-based Word Detector of [1] maps cluster of visemes to words through an iterative procedure.

**Figure 3:**
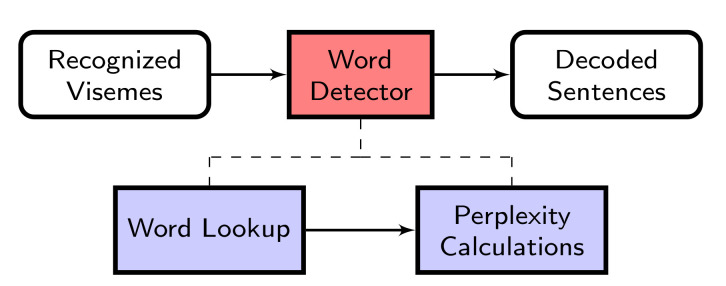
Processes of word detector.

Word matching is performed in different stages, shown in Figure 3, and the World Lookup stage is the very first stage. This is where every single cluster of visemes needs to be mapped to a set of words containing those visemes according to the mapping given by the Carnegie Mellon Pronouncing (CMU) Dictionary [35]. Once the word lookup stage is performed, the next stage of Word Detection is the Perplexity Calculations. The numerous possible choices of words that map to the visemes are combined, and perplexity iterations are performed to determine which combination of words is most likely to correspond to the uttered sentence, given the visemes recognised.

The word detector uses GPT to calculate perplexity by taking the exponential of the cross-entropy loss for a particular combination of words. Equations (20)–(22) below describe the relationship between perplexity PP, entropy *H*, and probability $P(w_{1},w_{2},\ldots ,w_{N})$ of a particular sequence of *N* words $\left(w_{1},w_{2},\ldots ,w_{N}\right)$ [46]. PP can expressed as the exponentiation of entropy *H* in Equation (20). The per-word entropy $\hat{H}$ is related to the probability $P(w_{1},w_{2},\ldots ,w_{N})$ of words $\left(w_{1},w_{2},\ldots ,w_{N}\right)$. The value of $P(w_{1},w_{2},\ldots ,w_{N})$ that results in the choice of words selected as the output is that which results in the minimisation of entropy in Equation (21), further resulting in the minimisation of perplexity provided in Equation (22) [46].

(20) $$PP=e^{H}$$

(21) $$\hat{H}=-\frac{1}{N}\ln P(w_{1},w_{2},\ldots ,w_{N})$$

(22) $$PP=P{\left(w_{1},w_{2},\ldots ,w_{N}\right)}^{-\frac{1}{N}}$$

When performing a conversion of visemes to words, some selection rules are implemented, as shown in Algorithm 1. If a viseme sequence has only one cluster matching to one word, that one word is selected as the output; whereas if a viseme sequence has only one cluster matching to several words, the word with largest expectation is selected as the output. This is determined by word rankings found in the Corpus of Contemporary American English (COCA) [36]. If a viseme sequence has more than one cluster, the words matching to the first two clusters are combined in every possible combination for the first iteration, and the combinations with the lowest 50 perplexity scores are kept. If there are more clusters in the sequence to be matched, then these combinations are in turn combined with the words matching with the next viseme cluster, keeping combinations with the lowest 50 perplexity scores at each iteration until the end of the sequence is reached. The selection of the lowest 50 perplexity scores at each iteration is based on an implementation of a local beam search with width 50.

One advantage of the language model of the GPT-based iterator is that when predicting a word at a particular timestep, it is able to base the prediction on all previous words predicted in the sentence. For a sentence of *K* words, the choice of the *K*th word can be conditioned on all the previous K−1 words as a context, which makes it a better implementation of Markov chains (Equation (23)). One disadvantage of this is that for long sentences, it would create more computational overhead, but it also makes the model more prone to errors if one word in the sentences is predicted incorrectly. The GPT-iterator calculates perplexity scores of words in combination; thus, one incorrect word causes cascading errors.

(23) $$P\left(w_{1},w_{2},\ldots ,w_{N}\right)=P\left(w_{1}\right)P\left(w_{2}|w_{1}\right)\ldots P\left(w_{i}|w_{1},w_{2},\ldots ,w_{i}-1\right)$$

**Algorithm 1:** Rules for Sentence Prediction**Require:** Viseme Clusters *V*, Beam With *B*, Coca Rankings *C*, Word Lexicon mapping *L*, Predicted Output *O*, Perplexity scores for sentences $p_{s}$ **if**
V.length=1 and $L_{V}.length=1$ **then**  Select 1 Word Match  $O\leftarrow L_{V}$ **if**
V.length=1 and $L_{V}.length>1$
**then**  Select Highest ranked word according to COCA  $O\leftarrow C^{-1}\left(\max\left\{C_{L}\right\}:w\right)$ **if**
V.length>1
**then**  Exhaustively combine words matching to $V_{n=0:1}$  Select Combinations with lowest *B* Perplexity scores for $V_{n=0:1}$  $p_{s}\leftarrow \min_{s\in B}\left\{s:PP(s)\right\}$  $sents\leftarrow p_{s}^{-1}\left(PP\left(s\right):s\right)$  **for** For n=2, n<V.length, n++
**do**   $L_{V}\leftarrow$ Perform word matches for $V_{n}$   Combine sentences from sents with words from $L_{V}$   Select Combinations with lowest *B* Perplexity scores   $p_{s}\leftarrow \min_{s\in B}\left\{s:PP(s)\right\}$   $sents\leftarrow p_{s}^{-1}\left(PP\left(s\right):s\right)$  $p_{s}\leftarrow \min\left\{s:PP(s)\right\}$  $O\leftarrow p_{s}^{-1}\left(PP\left(s\right):s\right)$


#### 3.3.4. Attention-GRU

Similarly to [20], the neural network architecture used for word detection follows a Recurrent Neural Network(RNN) Encoder-Decoder structure modelled according to neural machine translation whereby for a given input sequence of visemes *x*, a sequence of words *y* (Equation (24)) is outputted. However, the RNN here is in the form of a GRU not an LSTM; moreoveer, the input here takes the form a sequence of individual visemes as opposed to clusters of visemes and so only requires 17 tokens given in Table 3 to be encoded.

(24) $$y=\arg\max_{I\epsilon I^{*}}\left(y|x\right)$$

**Figure 4:**
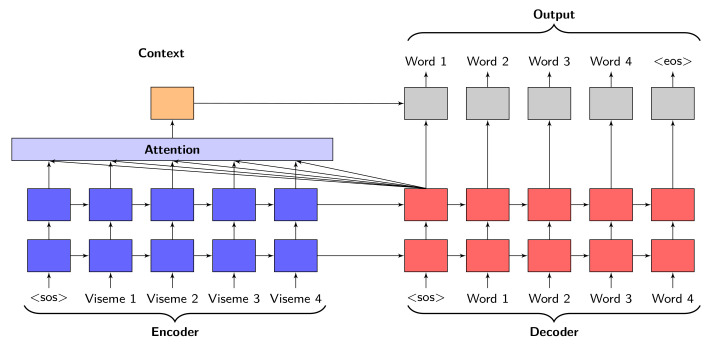
Components of Attention based GRU architecture.

An encoder-decoder framework (Figure 4) takes an input sequence of vectors $x=x_{1},\ldots ,x_{t}$ where $x_{t}$ corresponds to a vector and inputs into a vector *c* with hidden state $h_{t}$ at time *t*. The vector *c* is generated from the sequence of hidden states while *f* and *q* are non-linear variables. Vectors $h_{t}$ and *c* are provided in Equations (25) and (26) [47]. For this network, the encoder and decoder each consist of a GRU with 1024 nodes and a softmax layer with each possible word from the two corpuses LRS2 and LRS3 encoded as a class. Sequences of visemes are the sequence inputs and they consist of 17 input tokens.

(25) $$h_{t}=f\left(x_{t},h_{t-1}\right)$$

(26) $$c=q\left(\left\{h_{1},\ldots ,h_{t-1}\right\}\right)$$

The decoder is trained to predict the next word $y_{t}$ in a sequence given the context vector *c* and all the previously predicted words $y_{1},\ldots ,y_{t}$. The decoder defines a probability p(y) given in Equation (27) over the prediction probability p(y) by considering the joint conditional probability of all other previous words. A sentence predicted at time *t* follows with probability $p(y_{t})$ follows the expression given in Equation (28), where *g* is a nonlinear and $s_{t}$ is a the hidden state of the GRU.

(27) $$p\left(y\right)=\prod_{t=1}^{T}p\left(y_{t}|y_{1},\ldots ,y_{t},c\right)$$

(28) $$p\left(y_{t}|y_{1},\ldots ,y_{t},c\right)=g\left(y_{t-1},s_{t},c\right)$$

Sequences of visemes are inputted into the encoder, while teacher forcing is used to provide the inputs for the decoder (as seen in Figure 4). During training, the ground truth for the previous timesteps would be used as the decoder inputs, whereas for validation, the predicted outputs of the previous timesteps provide the inputs to the decoder.

The neural network architecture uses Bahdanau’s attention mechanism [47] for learning to align and predict sequences. The mechanism consist of components that include an alignment score, attention weights, and a context vector. The alignment score is a component for learning the mapping relationship between different inputs and outputs. The network is trained using identical hyper-parameters to the feed-forward neural networks and the same curriculum learning strategy.

The rationale behind the decision to use Bahdanau attention is arbitrary as the majority of neural machine translation model toolkits (which the proposed approach is modelled on) use Bahdanau attention, and the reported difference in performance between Bahdanau attention and Luong attention [48], for instance, has not been very significant.

One obvious advantage of this architecture is that it allows the overall speech recognition system to use fewer parameters (roughly 16 million parameters) in comparison to other lipreading systems such as the transformer based network of Afouras et el. [49] used for decoding sentences from LRS2, which used roughly 100 million parameters. Moreover, the GPT-based iterator for the viseme-to-word converter uses GPT to calculate perplexity for every word combination made at each stage of the iterative procedure, meaning that the number of times the model will have to be evaluated will increase exponentially with the number of words contained in an uttered sentence. The iterator uses a beam search width of 50; thus, a minimum of $50^{n-1}$ perplexity iterations would need to be performed for a sentence with *n* words.

### 3.4. Data Noisification

Data noisification [50] is implemented for the purpose of evaluating how robust the viseme-to-word classifier is to errancy in the inputted visemes by adding noise in the form of misclassification to the inputs. Noisification is implemented by adding small perturbations to the input visemes, and there are four different techniques being implemented. These four techniques include random deletion, insertion, substitution, and swapping [50].

Random Deletion [50] is a technique where random visemes are deleted according to a probabilistic metric $\alpha_{rd}$. The total number of visemes $n_{r}d$ that is deleted for a sequence with $n_{v}$ total visemes is the equivalent product of $\alpha_{rd}$ and $n_{v}$ rounded to the nearest integer given in Equation (29).

(29) $$n_{rd}=\alpha_{rd}n_{v}$$

Random Swapping [50] involves the swapping of random visemes implemented according to a probabilistic metric $\alpha_{rs}$ and the total number of visemes $n_{v}$. The number of swap operations $n_{r}s$ that takes place is governed by the outcome of Equation (30). This is simply the product of $\alpha_{rs}$ and $n_{v}$ rounded to the nearest integer. The two visemes that become swapped are chosen by generating two random numbers to determine the positions of the two respective visemes to be swapped.

(30) $$n_{rs}=\alpha_{rs}n_{v}$$

Random insertion [50] is a process where random visemes are inserted along parts of the viseme sequences according to probabilistic metric $\alpha_{rs}$ and the number of visemes $n_{v}$. Similarly to random swapping, the number of insertion operations to be performed is calculated by using a similar equation. The number of insertions $n_{r}i$ that occurs is governed by the outcome of Equation (31), which is the product of $\alpha_{ri}$ and $n_{v}$ rounded to the nearest integer.

(31) $$n_{ri}=\alpha_{ri}n_{v}$$

**Figure 5:**
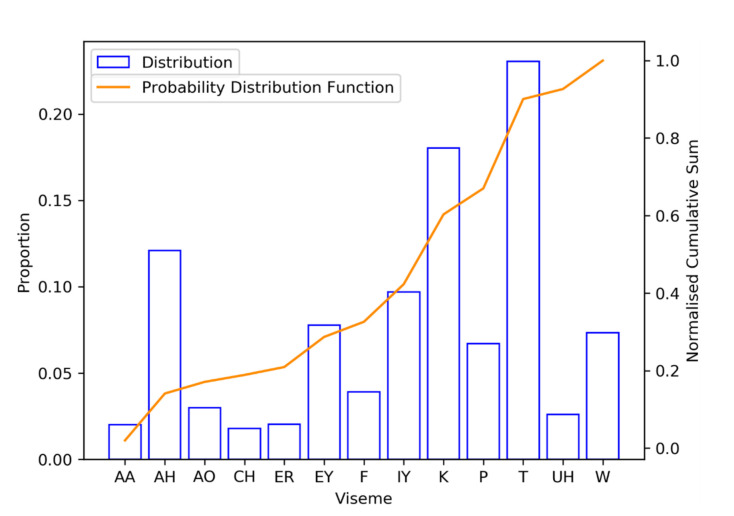
Probability distribution for generating visemes.

The choice of a viseme that does is inserted for the random insertion operation is determined by a random number operation such that the identity of the viseme will be generated according to a probability distribution matching the viseme distribution of LRS2 corpus. The rationale behind this is that when visemes are misclassified, they are most likely to be classified as any of the most frequently appearing visemes found in the training set. Figure 5 shows the cumulative probability distribution for visemes contained within the LRS2 training set.

The other technique called Random Substitution [50] is where random positions along the viseme sequence are chosen, and the viseme corresponding to that position is substituted for another viseme. The number of substitutions that takes place is set by Equation (32) where for $n_{v}$ total visemes and a probabilistic metric $\alpha_{sr}$ for substitution, a number of substitutions operations $n_{sr}$ take place. Similarly to the random insertion operation, the new viseme being substituted will be generated according to a probability distribution matching the viseme distribution of LRS2 training set.

(32) $$n_{sr}=\alpha_{sr}n_{v}$$

### 3.5. Systems Performance Measures

The measures used to evaluate the viseme-to-word conversion are edit distance-based metrics and are computed by calculating the normalized edit distance between ground truth and the predicted sentence. Metrics reported in this paper include Viseme Character Error Rate (CER), Word Error Rates (WER), and Sentence Accuracy Rate (SAR).

Error rate metrics used for evaluating accuracy are given by calculating the overall edit distance. In order to determine misclassifications, one has to compare decoded speech to actual speech. The general expression for Error Rate (ER) is given in Equation (33) where *N* is the total number of characters in the actual speech, *S* is the number of characters substituted for wrong classifications, *I* is the number of characters inserted for those not picked up, and *D* is the number of deletions made for decoded characters that should not be present. VER, CER, and WER are all calculated in this manner with the expressions given in Equations (34)–(36), where *V*, *C*, and *W* correspond to either characters, words, and visemes being substituted, deleted, or inserted.

(33) $$ER=\frac{S+D+I}{N}$$

(34) $$VER=\frac{V_{S}+V_{D}+V_{I}}{V_{N}}$$

(35) $$CER=\frac{C_{S}+C_{D}+C_{I}}{C_{N}}$$

(36) $$WER=\frac{W_{S}+W_{D}+W_{I}}{W_{N}}$$

The Sentence Accuracy Rate is a binary metric that takes the value of one if the predicted sentence $P_{P}$ is equal to the ground truth $P_{T}$; otherwise, it takes the value of zero (Equation (37)).

(37) $$SAR=\left\{\begin{array}{cc} 1, & P_{P}=P_{T}\\ 0, & P_{P}\neq P_{T} \end{array}\right.$$

## 4. Experiment and Results

For the training and evaluation of the viseme-to-word converters mentioned in Section 3 excluding the GPT-based interator, LRS2 sentence data described in Section 3.1 have been used with 80% of all sentences used for training (37,666 samples) and 20% of sentences being utilised for testing (9416 samples). *k*-Fold cross validation has been used with a fold value for *k* = 5, and for each fold, a different set of 9416 samples were used. Viseme-to-word conversion has also been performed for sentences from the LRS3 corpus with 26,588 samples for training, 6477 samples for testing, and *k* = 5 for *k*-Fold cross validation. All neural network simulations were implemented in TensorFlow and trained on a single GeForce GTX 1080 Ti GPU with 11 GB memory.

The metrics reported include CER, WER, SAR, and word accuracies (WAR). Performance results for word prediction are given for the following three situations: (1) correct visemes, (2) visemes classified as outputs of the viseme classifier reported in [1], and (3) perturbed visemes with added noise to vary the errancy. For Situation 1, the final performance results reported are averaged over each of the folds for the k-Fold cross validation. However, for situations two and three, model k-Fold old one was used for both the LRS2 and LRS3 corpuses; thus, the results were reported for that particular fold.

Situation 2 is significant because it is identical to substituting the viseme-to-word converter used in [1] with the GRU-based converter proposed in this paper, whilst using the same viseme classifier for classifying visemes.

For the third situation, the accuracy of incident visemes is altered by using the noisification process described in Section 3.4 where all probabilistic indicators are modified to vary viseme accuracy. The probabilistic indicators $\alpha_{rd}$, $\alpha_{ri}$, $\alpha_{rs}$, and $\alpha_{sr}$ for deletion, insertion, swapping, and substitution, respectively, are all set to the same value $\alpha_{mod}$ and incremented to vary the noise level on the visemes being inputted. Once the viseme-to-word detector has been trained, the trained network is evaluated on different incident viseme accuracies ranging from 70% to 100% to examine its robustness to noise.

The GRU architecture, feed-forward network and HMM were trained for several epochs until no improvements in either the training or validation losses were observed. It was at the point that the validation loss stopped converging that the performance of the model was evaluated. In addition to modelling the GRU network’s performance under different levels of viseme noise, it has also been compared with the performance of the GPT-based iterator.

Table 4 and Table 5 lists the performance metrics of all four models for Situation 1 for the LRS2 and LRS3 corpuses when inputted visemes are known to be 100% correct. It it is noticeable that the performances of all four models when decoding sentences from the LRS3 set were not as good as those for LRS2, and this can be explained by the fact that the LRS3 corpus consists of longer sentences and a deviation between the predicted sentence, and ground truth becomes more likely as the sentence lengths increase.

Table 6 gives the performance metrics of the four architectures for Situation 2 using the output of the viseme classifier in [1] where VER≈4 %, and it is clear that the Attention based GRU and GPT-based iterators are significantly more effective in their conversion compared with the feed-forward network and HMM because they are able to exploit larger context windows.

**Figure 6:**
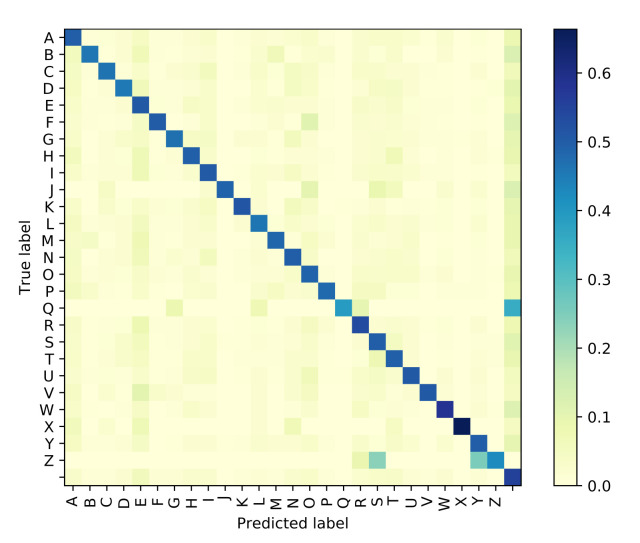
Confusion Matrix for GPT-based Iterator.

**Figure 7:**
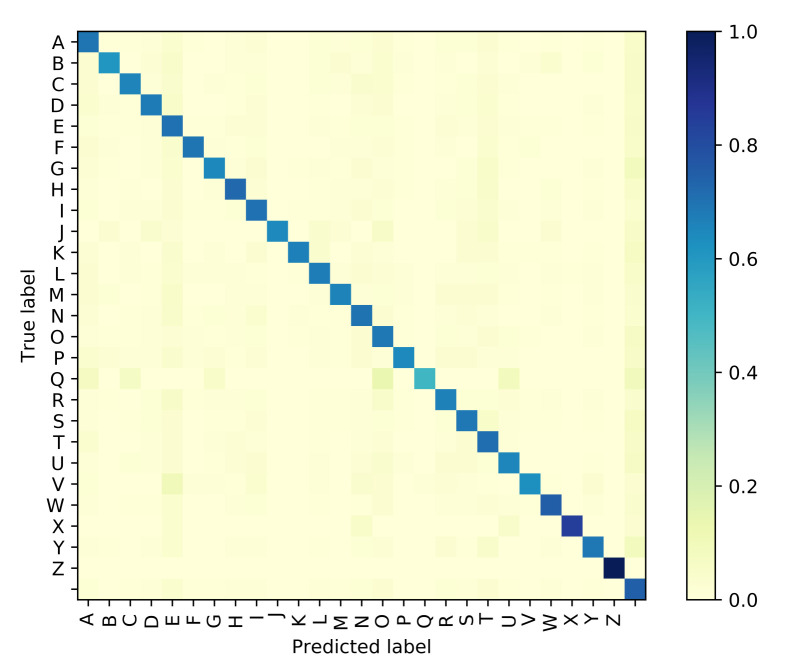
Confusion Matrix for Attention-based GRU.

**Figure 8:**
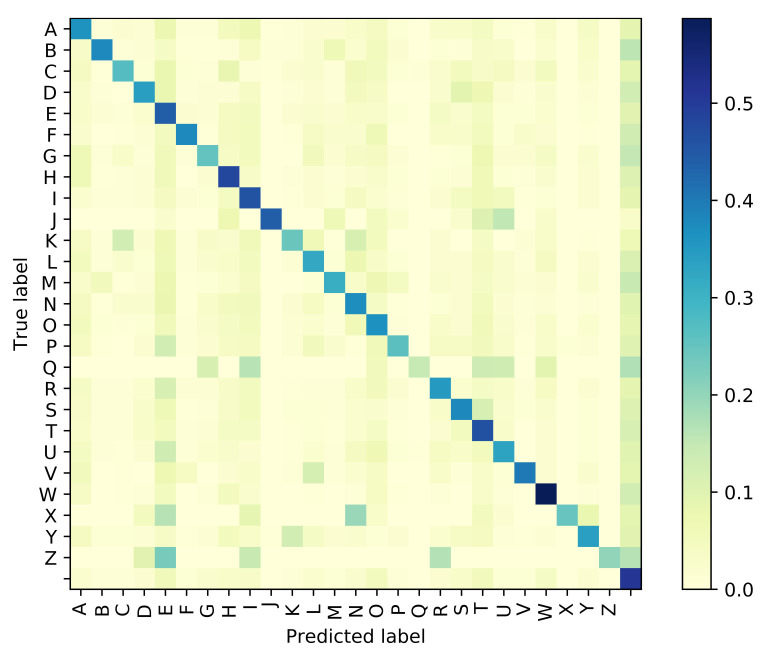
Confusion Matrix for Feed-Forward Network.

**Figure 9:**
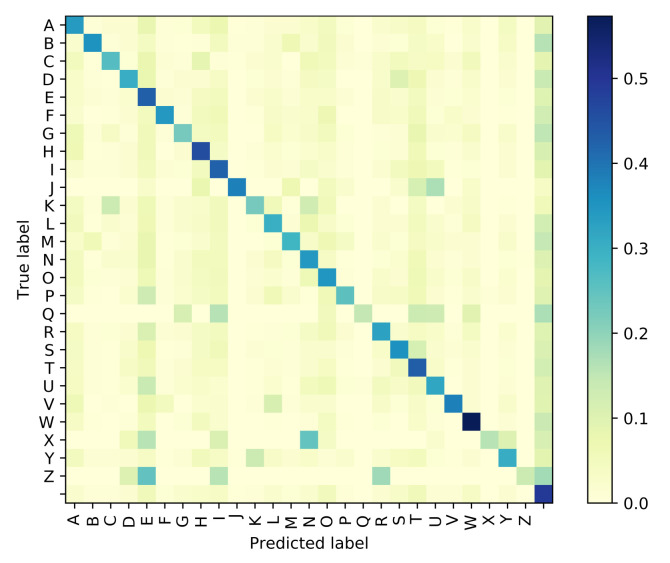
Confusion Matrix for Hidden Markov Model.

The Attention based GRU outperforms the GPT-based iterator for Situation 1 where the identity of visemes is known with 100% accuracy. However, even with the smallest noise added to viseme inputs, the difference between the performances of the two models diverge, and the GRU network is clearly more resilient to perturbations in the input viseme sequences. Tables 9 and 10 both provide samples of how some sentences are predicted by all four models along with time elapsed for execution. Confusion matrices have been plotted in Figure 6, Figure 7, Figure 8 and Figure 9 for the GPT-based iterator, GRU network, Feed-forward network, and HMM correspondingly.

**Figure 10:**
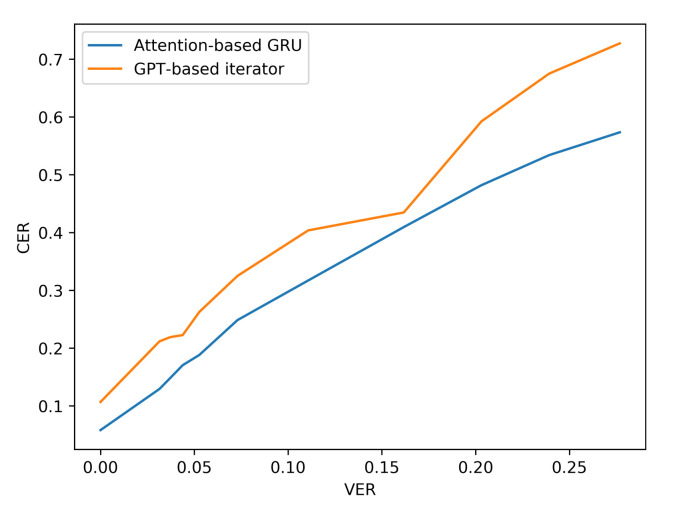
CER performance under varying noise levels (evaluation on LRS2 corpus).

**Figure 11:**
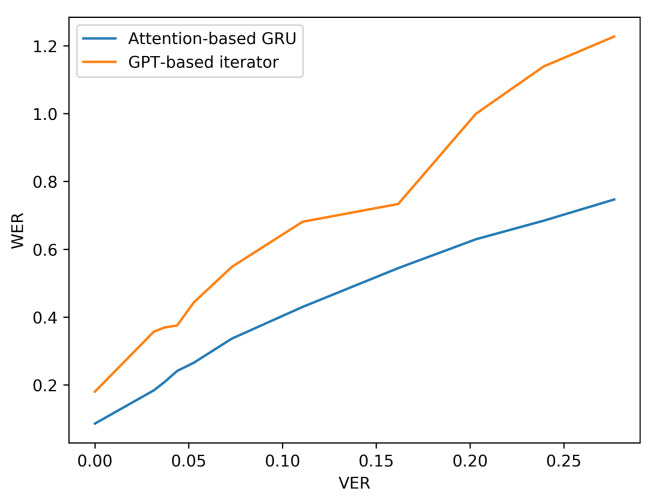
WER performance under varying noise levels (evaluation on LRS2 corpus).

**Figure 12:**
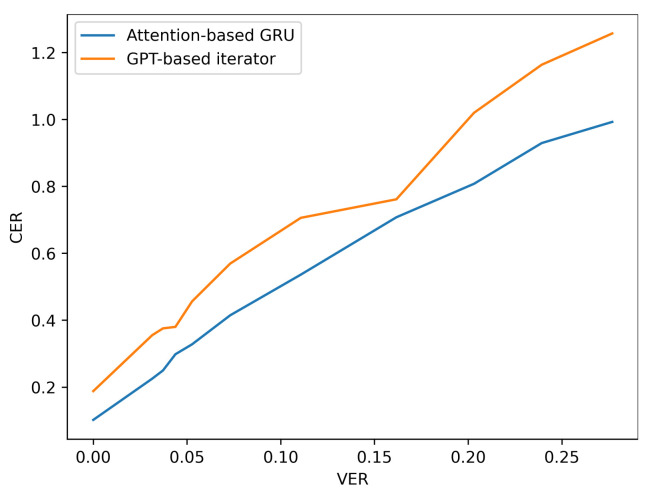
CER performance under varying noise levels (evaluation on LRS3 corpus).

**Figure 13:**
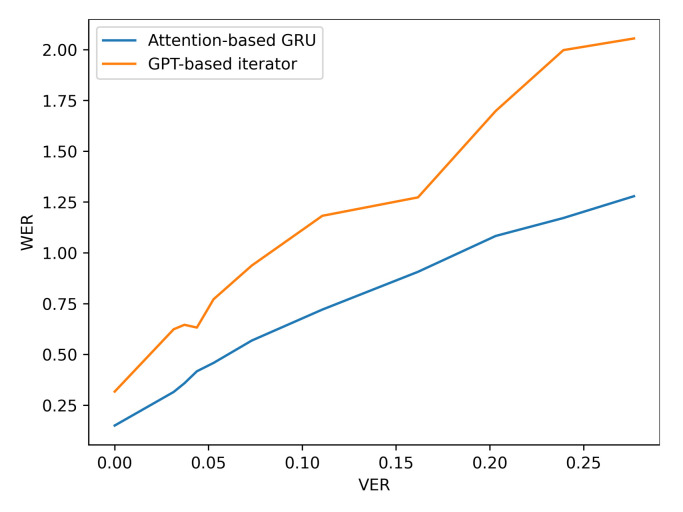
WER performance under varying noise levels (evaluation on LRS3 corpus).

Additionally, the resilience of the Attention based GRU to perturbations compared with the GPT-transformer based iterator is further observed when more noise is added to the input viseme sequences by analysing the performance results of situation three. The difference in character and word error rates recorded by both models grows even further apart with the increase in errancy of visemes as shown in Table 7 and Figure 10 and Figure 11 (for LRS2) or Table 8 and Figure 12 and Figure 13 (for LRS3).

The improvement in performance of predicting sentences with the GRU network especially with perturbed inputs can be explained by two main factors. The first is that word matching is performed on an individual viseme level rather than being performed on a cluster level such as for the perplexity-based iterator; thus, if there is a word with one viseme being decoded incorrectly, the word it is contained in can still be identified correctly because the network is designed to classify visemes in combination.

This is not the case for the GPT-based iterator that maps clusters to words, meaning that one viseme being decoded incorrectly would cause the entire cluster to be matched to the wrong words. An example of this can be observed with the sentence “for a brief time” being decoded as “or a brief time” by the GPT-based iterator. The reason for this incorrect prediction is that the first viseme “F” has been incorrectly decoded as “AO”, yet the Attention based GRU is able to predict the spoken sentence correctly.

The second reason for there being a better resilience is that the GRU network is better at modelling shorter groups of words [51]. It does not suffer from the problem posed in the mapping of viseme clusters to words using the GPT-based iterator whereby compound errors occur in the combination of words during the iterations and in which the sentence being decoded is based on the conditional dependence of word combinations.

The GPT-iterator model uses GPT to calculate perplexity scores of word combinations matching to viseme clusters in an iterative manner starting from the beginning of the sentence as opposed to being used for word prediction. If one viseme is misclassified, the input cluster would then be wrong, resulting in incorrect word matches not only for that one cluster but would also cause words further along the sequence to be incorrectly predicted because the words in the rest of the sentence are all dependent on words that have been previously predicted. Moreover, due to the curriculum learning strategy deployed for training the GRU network, it is better at recognising shorter N-grams [52,53,54].

When looking at the differences in how some sentences were decoded by both systems, it is clear that the system with the GRU network is less affected by compound errors in the prediction because when one word has been predicted incorrectly, it will be less likely that other words in the outputted sentence would also be classified incorrectly too.

In addition to GRU network being more robust to noisy inputs than the GPT-based iterator, it is also more efficient and requires less overhead, which is why it takes significantly less time to execute than the GPT-based iterator. The GPT-based iterator uses approximately 11 times the number of parameters as the GRU network does, and as observed in Table 9, it takes significantly more time when decoding visemes.

When comparing the conversion of sequences of visemes to words for all four models for some samples in Table 9 and Table 10, it is noticeable that the accuracies of the two models utilising unlimited context, namely the GPT-based Iterator and Attention based GRU, are significantly more accurate in their conversions compared with both the Feed-Forward network and Hidden Markov Model that utilise fixed-context windows. For instance, for the sequence of visemes that corresponds to the sentence “for a brief time”, the last viseme cluster corresponding to the word “time” was actually predicted by both the Feed-Forward Network and Hidden Markov Model as “type”. The words “time” and “type” are both homopheme words, yet they are both semantically different, and a longer context window is needed to be able to exploit semantic information to predict the correct word.

## 5. Conclusions

A viseme-to-word conversion model has been proposed that is robust, quick to execute, and effective at discriminating between words that share identical visemes. Its performance has been compared with three other conversion model approaches. The model has been proven to be effective at disambiguating between words that are semantically and syntactically different as well as being able to model long and short term dependencies in ordr to render it robust to incorrectly classified visemes. The converter’s robustness has been verified on the LRS2 and LRS3 corpuses, and when implemented in a neural network-based architecture for lip reading sentences from the LRS2 dataset, a 79.6% word accuracy rate was recorded—an improvement of 15.0% from the previous state of the art.

Future research includes improving the robustness of viseme-to-word conversion further by using techniques such as augmentation in the training phase. Moreover, there are other types of networks that could be used to enhance the overall word accuracy further such as bidirectional RNNs as these can exploit right-to-left context in addition to left-to-right context for word prediction. There is also merit in considering the use of either Attention-Transformers or Temporal Convolutional Networks as conversion models because they can process inputs in parallels as opposed to RNNs, which process inputs sequentially.

It would also be ideal if it was possible to exploit knowledge regarding words that either consist or do not consist of unique visemes sequences, as has been conducted for the case of viseme-to-word conversion when the identities of the inputted visemes are known with absolute precision.

## Figures and Tables

**Table 1:** Two-stage speech recognition approaches where CI and CD refer to context-independent and context-dependent models, and SAT referss to speaker adaptive training.

Approach	Viseme Format	1st Stage Feature Extractor	First Stage Classifier	Second Stage Classifier	Dataset	Unit Classif. Acc. (%)	Word Classif. Acc. (%)
Lan and Harvey [5]	Bigram	LDA + PCA	HMM-GMM	HMM	LiLiR	45.67	14.08
Almajai [6]	Bigram	LDA HMM	HMM	HMM	LiLiR	-	17.74
Almajai [6]	Bigram	LDA+MLLT	HMM	HMM	LiLiR	-	22.82
Almajai [6]	Bigram	LDA+MLLT+SAT	HMM	HMM	LiLiR	-	37.71
Almajai [6]	Bigram	LDA+MLLT+SAT	HMM	Feed-forward	LiLiR	-	47.75
Bear and Harvey [4]	Bigram	Active Appearance Model	HMM	HMM	LiLiR	8.51	4.38
Thangthai [3]	Bigram	Discrete Cosine Transform	CD-GMM + SAT	WFST	TCD-TIMIT	42.48	10.47
Thangthai [3]	Bigram	Discrete Cosine Transform	CD-DNN	WFST	TCD-TIMIT	38.00	9.17
Thangthai [3]	Bigram	Eigenlips	CD-GMM + SAT	WFST	TCD-TIMIT	44.61	12.15
Thangthai [3]	Bigram	Eigenlips	CD-DNN	WFST	TCD-TIMIT	44.60	19.15
Howell [2]	Bigram	Active Appearance Model	CD-HMM	HMM	RM-3000	52.31	43.47
Fenghour [20]	Cluster	N/A	N/A	Encoder-Decoder LSTM	LRS2	N/A	72.20
Fenghour [1]	Cluster	ResNet CNN	Linear Transformer	GPT-Transformer based Iterator	LRS2	95.40	64.60

**Table 2:** A sequence of visemes and its corresponding word match.

**Visemes**	<sos>	’T’	’AH’	<space>	’T’	’ER’	’P’	’W’	’AH’	’T’	<space>	’W’	’AA’	’T’	<eos>
**Words**	<sos>	“THE”			“SURPRISE”							“WAS”			<eos>

**Table 3:** Viseme Classes used for input to viseme-to-word converter [36].

[pad], AA, AH, AO, CH, ER, EY, F, IY, K, P, T, UH, W, <sos>, <eos>, [space]

**Table 4:** Performance of viseme-to-word converters for Situation 1 on the LRS2 dataset.

Converter	Fold No.	CER (%)	WER (%)	SAR (%)	WAR (%)
GPT-based Iterator	Fold 1	10.7	18.0	56.8	82.0
GPT-based Iterator	Fold 2	11.4	19.5	55.1	80.5
GPT-based Iterator	Fold 3	11.2	19.1	54.8	80.9
GPT-based Iterator	Fold 4	12.0	20.3	54.2	79.7
GPT-based Iterator	Fold 5	11.0	18.6	54.9	81.4
**GPT-based Iterator**	**Average**	**11.3 ± 0.5**	**19.1 ± 0.9**	**55.2 ± 1.0**	**80.9 ± 0.9**
Attention-based GRU	Fold 1	6.2	8.8	74.9	91.2
Attention-based GRU	Fold 2	6.9	9.7	74.2	90.3
Attention-based GRU	Fold 3	7.5	10.6	73.4	89.4
Attention-based GRU	Fold 4	7.4	10.4	73.4	89.6
Attention-based GRU	Fold 5	7.1	10.2	73.8	89.8
**Attention-based GRU**	**Average**	**7.0 ± 0.5**	**9.9 ± 0.7**	**73.9 ± 0.6**	**90.1 ± 0.7**
Feed-Forward Network	Fold 1	31.7	42.7	9.4	57.3
Feed-Forward Network	Fold 2	32.4	43.4	8.6	56.6
Feed-Forward Network	Fold 3	33.0	44.1	7.8	55.9
Feed-Forward Network	Fold 4	32.8	43.9	8.1	56.1
Feed-Forward Network	Fold 5	32.6	43.5	8.1	56.5
**Feed-Forward Network**	**Average**	**32.5 ± 0.5**	**43.5 ± 0.5**	**8.4 ± 0.6**	**56.5 ± 0.5**
Hidden Markov Model	Fold 1	34.0	44.5	9.0	55.5
Hidden Markov Model	Fold 2	35.3	46.2	7.8	53.8
Hidden Markov Model	Fold 3	36.1	49.8	6.5	50.2
Hidden Markov Model	Fold 4	35.8	48.0	8.0	52.0
Hidden Markov Model	Fold 5	35.2	45.9	7.4	54.1
**Hidden Markov Model**	**Average**	**35.3 ± 0.8**	**46.9 ± 2.1**	**7.7 ± 0.9**	**53.1 ± 2.1**

**Table 5:** Performance of viseme-to-word converters for Situation 1 on the LRS3 dataset.

Converter	Fold No.	CER (%)	WER (%)	SAR (%)	WAR (%)
GPT-based Iterator	Fold 1	18.8	31.7	36.2	68.3
GPT-based Iterator	Fold 2	19.7	32.5	34.8	67.5
GPT-based Iterator	Fold 3	20.3	33.3	33.7	66.7
GPT-based Iterator	Fold 4	19.4	32.2	35.3	67.8
GPT-based Iterator	Fold 5	18.6	31.3	36.3	68.7
**GPT-based Iterator**	**Average**	**19.4 ± 0.7**	**32.2 ± 0.8**	**35.3 ± 1.1**	**67.8 ± 0.8**
Attention-based GRU	Fold 1	10.2	15.0	59.2	85.0
Attention-based GRU	Fold 2	10.5	15.4	59.0	84.6
Attention-based GRU	Fold 3	11.2	16.1	58.2	83.9
Attention-based GRU	Fold 4	10.9	15.8	58.2	84.2
Attention-based GRU	Fold 5	11.5	16.8	57.6	83.2
**Attention-based GRU**	**Average**	**10.9 ± 0.5**	**15.8 ± 0.7**	**58.4 ± 0.7**	**84.2 ± 0.7**
Feed-Forward Network	Fold 1	38.5	49.9	7.1	50.1
Feed-Forward Network	Fold 2	39.4	51.3	6.3	48.7
Feed-Forward Network	Fold 3	41.1	52.1	5.4	47.9
Feed-Forward Network	Fold 4	39.6	51.6	6.2	48.4
Feed-Forward Network	Fold 5	39.3	51.3	6.3	48.7
**Feed-Forward Network**	**Average**	**39.6 ± 0.9**	**51.2 ± 0.8**	**6.3 ± 0.6**	**48.8 ± 0.8**
Hidden Markov Model	Fold 1	41.3	52.1	7.0	47.9
Hidden Markov Model	Fold 2	42.5	54.2	6.1	45.8
Hidden Markov Model	Fold 3	43.3	54.9	5.4	45.1
Hidden Markov Model	Fold 4	42.6	54.4	5.8	45.6
Hidden Markov Model	Fold 5	42.2	53.8	6.3	46.2
**Hidden Markov Model**	**Average**	**42.4 ± 0.7**	**53.9 ± 1.1**	**6.1 ± 0.6**	**46.1 ± 1.1**

**Table 6:** Performance of viseme-to-word converters for Situation 2.

Viseme-to-Word Converter	CER (%)	WER (%)	SAR (%)	WAR (%)
GPT-based iterator	23.1	35.4	33.4	64.6
Attention-based GRU	14.0	20.4	49.8	79.6
Feed-Forward Network	67.2	78.7	2.9	21.3
Hidden Markov Model	71.4	81.7	2.8	18.3

**Table 7:** Performance of viseme-to-word converters under varying noise levels on the LRS2 dataset.

$\alpha_{\mathrm{mod}}$	VER (%)	Attention-Based GRU		GPT-Based Iterator	
		CER (%)	WER (%)	CER (%)	WER (%)
0	0.0	5.8	8.6	10.7	18.0
5	3.1	12.9	18.4	21.2	35.7
6	3.7	14.8	20.9	21.9	37.0
7	4.4	17.1	24.1	22.3	37.5
8	5.3	18.8	26.5	26.3	44.3
10	7.3	24.9	33.7	32.5	54.8
15	11.1	31.7	43.0	40.4	68.1
20	16.2	40.9	54.4	43.5	73.4
25	20.3	48.2	63.0	59.2	100.0
30	23.9	53.4	68.4	67.5	113.9
35	27.7	57.2	74.5	72.7	122.7

**Table 8:** Performance of viseme-to-word converters under varying noise levels on the LRS3 dataset.

$\alpha_{\mathrm{mod}}$	VER (%)	Attention-Based GRU		GPT-Based Iterator	
		CER (%)	WER (%)	CER (%)	WER (%)
0	0.0	10.2	15.0	18.8	31.7
5	2.8	22.5	31.5	35.5	62.4
6	3.5	25.0	35.8	37.6	64.6
7	4.5	29.8	41.7	38.0	63.2
8	5.2	32.8	45.8	45.6	77.1
10	7.6	41.5	56.8	56.9	93.7
15	11.0	53.6	72.1	70.6	118.2
20	16.5	70.8	90.7	76.1	127.3
25	20.1	80.8	108.3	102.0	169.8
30	23.9	92.9	117.1	116.4	199.8
35	27.5	99.3	127.9	125.7	205.5

**Table 9:** Examples of decoded sentences from the two viseme-to-word converters.

Actual Subtitle	Actual Visemes	Predicted Visemes	GPT-Based Iterator		Attention-Based GRU	
			Decoded Subtitle	Execution Time (s)	Decoded Subtitle	Execution Time (s)
WHEN THERE ISN’T MUCH ELSE IN THE GARDEN	(’W’, ’EY’, ’K’), (’T’, ’EY’, ’W’), (’IY’, ’T’, ’AH’, ’K’, ’T’), (’P’, ’AH’, ’CH’), (’EY’, ’K’, ’T’), (’IY’, ’K’), (’T’, ’AH’), (’K’, ’AA’, ’W’, ’T’, ’AH’, ’K’)	(’W’, ’EY’, ’K’), (’T’, ’EY’, ’W’), (’IY’, ’T’, ’AH’, ’K’, ’T’), (’P’, ’AH’, ’CH’), (’EY’, ’K’, ’T’), (’IY’, ’K’), (’T’, ’AH’), (’K’, ’AA’, ’W’, ’T’, ’AH’, ’K’)	WHEN THERE ISN’T MUCH ELSE IN THE GARDEN	147.35	WHEN THEY’RE ISN’T MUCH ELSE IN THE GARDEN	0.08
SORT OF SECOND HALF OF OCTOBER	(’T’, ’AO’, ’W’, ’T’), (’AH’, ’F’), (’T’, ’EY’, ’K’, ’AH’, ’K’, ’T’), (’K’, ’EY’, ’F’), (’AH’, ’F’), (’AA’, ’K’, ’T’, ’AO’, ’P’, ’ER’)	(’T’, ’AO’, ’W’, ’T’), (’AH’, ’F’), (’T’, ’EY’, ’K’, ’AH’, ’K’, ’T’), (’K’, ’EY’, ’F’), (’AH’, ’F’), (’AA’, ’K’, ’T’, ’AO’, ’P’, ’ER’)	SORT OF SECOND HALF OF OCTOBER	32.86	SORT OF SECOND HALF OF OCTOBER	0.05
WELL INTO NOVEMBER	(’W’, ’EY’, ’K’), (’IY’, ’K’, ’T’, ’UH’), (’K’, ’AO’, ’F’, ’EY’, ’P’, ’P’, ’ER’)	(’W’, ’EY’, ’K’), (’IY’, ’K’, ’T’, ’UH’), (’K’, ’AO’, ’F’, ’EY’, ’P’, ’P’, ’ER’)	RAN INTO NOVEMBER	2.83	WELL INTO NOVEMBER	0.03
WE CAN JUST ABOUT GET AWAY WITH IT NOW	(’W’, ’IY’), (’K’, ’EY’, ’K’), (’CH’, ’AH’, ’T’, ’T’), (’AH’, ’P’, ’EY’, ’T’), (’K’, ’EY’, ’T’), (’AH’, ’W’, ’EY’), (’W’, ’IY’, ’T’), (’IY’, ’T’,) (’K’, ’EY’)	(’W’, ’IY’), (’K’, ’EY’, ’K’), (’CH’, ’AH’, ’T’, ’T’), (’AH’, ’P’, ’EY’, ’T’), (’K’, ’EY’, ’T’), (’AH’, ’W’, ’EY’), (’W’, ’IY’, ’T’), (’IY’, ’IY’), (’K’, ’EY’)	WE CAN JUST ABOUT GET AWAY WITH IIE KAYE	323.84	WE CAN JUST ABOUT GET AWAY WITH IT NOW	0.07
AND IF YOU WANT WONDERFUL	(’AH’, ’K’, ’T’), (’IY’, ’F’), (’K’, ’UH’), (’W’, ’AA’, ’K’, ’T’), (’W’, ’AH’, ’K’, ’T’, ’ER’, ’F’, ’AH’, ’K’)	(’AH’, ’K’, ’T’), (’IY’, ’F’), (’K’, ’UH’), (’W’, ’AA’, ’K’, ’T’), (’W’, ’AH’, ’K’, ’T’, ’ER’, ’F’, ’AH’, ’K’)	AND IF YOU WANT WONDERFUL	35.19	AND IF YOU WANT WONDERFUL	0.05
FOR A BRIEF TIME	(’F’, ’AO’, ’W’), (’AH’), (’P’, ’W’, ’IY’, ’F’), (’T’, ’AH’, ’P’)	(’AO’, ’AO’, ’W’), (’AH’), (’P’, ’W’, ’IY’, ’F’), (’T’, ’AH’, ’P’)	OR A BRIEF TIME	21.95	FOR A BRIEF TIME	0.05
IT WILL CHANGE LIVES	(’IY’, ’T’), (’W’, ’IY’, ’K’), (’CH’, ’EY’, ’K’, ’CH’), (’K’, ’IY’, ’F’, ’T’)	(’T’, ’T’), (’W’, ’IY’, ’K’), (’CH’, ’EY’, ’K’, ’CH’), (’K’, ’IY’, ’F’, ’T’)	THS WE’LL CHANGE LIFFE’S	27.53	THIS WILL CHANGE LIVES	0.05
I THINK IT’S BRILLIANT	(’AH’), (’T’, ’IY’, ’K’, ’K’), (’IY’, ’T’, ’T’), (’P’, ’W’, ’IY’, ’K’, ’K’, ’AH’, ’K’, ’T’)	(’AH’), (’T’, ’IY’, ’K’), (’IY’, ’T’, ’T’), (’P’, ’W’, ’IY’, ’K’, ’K’, ’AH’, ’K’, ’T’)	EYE ’TIL IT’S PRINGLE’S	13.24	I THING IT’S BRILLIANT	0.05
BUT IT’S A DECENT SIZE	(’P’, ’AH’, ’T’), (’IY’, ’T’, ’T’), (’AH’), (’T’, ’IY’, ’T’, ’AH’, ’K’, ’T’), (’T’, ’AH’, ’T’)	(’P’, ’AH’, ’T’), (’IY’, ’T’, ’T’), (’AH’), (’T’, ’IY’, ’T’, ’AH’, ’K’, ’T’), (’T’, ’AH’, ’T’)	BUT IT’S I DIDN’T SUSS	79.88	BUT IT’S A DECENT SIZE	0.06

**Table 10:** Examples of decoded sentences from the two viseme-to-word converters.

Actual Subtitle	Actual Visemes	Predicted Visemes	Hidden Markov Model		Feed-Forward Network	
			Decoded Subtitle	Execution Time (s)	Decoded Subtitle	Execution Time (s)
WHEN THERE ISN’T MUCH ELSE IN THE GARDEN	(’W’, ’EY’, ’K’), (’T’, ’EY’, ’W’), (’IY’, ’T’, ’AH’, ’K’, ’T’), (’P’, ’AH’, ’CH’), (’EY’, ’K’, ’T’), (’IY’, ’K’), (’T’, ’AH’), (’K’, ’AA’, ’W’, ’T’, ’AH’, ’K’)	(’W’, ’EY’, ’K’), (’T’, ’EY’, ’W’), (’IY’, ’T’, ’AH’, ’K’, ’T’), (’P’, ’AH’, ’CH’), (’EY’, ’K’, ’T’), (’IY’, ’K’), (’T’, ’AH’), (’K’, ’AA’, ’W’, ’T’, ’AH’, ’K’)	WHEN THEY’RE ISN’T BE JUST IN THE GARDEN	0.07	WHEN THEY’RE ISN’T JUST BE IN THE GARDEN	0.08
SORT OF SECOND HALF OF OCTOBER	(’T’, ’AO’, ’W’, ’T’), (’AH’, ’F’), (’T’, ’EY’, ’K’, ’AH’, ’K’, ’T’), (’K’, ’EY’, ’F’), (’AH’, ’F’), (’AA’, ’K’, ’T’, ’AO’, ’P’, ’ER’)	(’T’, ’AO’, ’W’, ’T’), (’AH’, ’F’), (’T’, ’EY’, ’K’, ’AH’, ’K’, ’T’), (’K’, ’EY’, ’F’), (’AH’, ’F’), (’AA’, ’K’, ’T’, ’AO’, ’P’, ’ER’)	SORT I’VE SECOND HALF I’VE WHICH	0.04	SORT I SECOND HALF OF OCTOBER	0.05
WELL INTO NOVEMBER	(’W’, ’EY’, ’K’), (’IY’, ’K’, ’T’, ’UH’), (’K’, ’AO’, ’F’, ’EY’, ’P’, ’P’, ’ER’)	(’W’, ’EY’, ’K’), (’IY’, ’K’, ’T’, ’UH’), (’K’, ’AO’, ’F’, ’EY’, ’P’, ’P’, ’ER’)	WHEN INTO NOVEMBER	0.03	WHEN INTO NOVEMBER	0.04
WE CAN JUST ABOUT GET AWAY WITH IT NOW	(’W’, ’IY’), (’K’, ’EY’, ’K’), (’CH’, ’AH’, ’T’, ’T’), (’AH’, ’P’, ’EY’, ’T’), (’K’, ’EY’, ’T’), (’AH’, ’W’, ’EY’), (’W’, ’IY’, ’T’), (’IY’, ’T’,) (’K’, ’EY’)	(’W’, ’IY’), (’K’, ’EY’, ’K’), (’CH’, ’AH’, ’T’, ’T’), (’AH’, ’P’, ’EY’, ’T’), (’K’, ’EY’, ’T’), (’AH’, ’W’, ’EY’), (’W’, ’IY’, ’T’), (’IY’, ’IY’), (’K’, ’EY’)	WE CAN JUST ABOUT GET AWAY WITH IT HOW	0.07	WE CAN JUST ABOUT GET AWAY WITH IT NOW	0.07
AND IF YOU WANT WONDERFUL	(’AH’, ’K’, ’T’), (’IY’, ’F’), (’K’, ’UH’), (’W’, ’AA’, ’K’, ’T’), (’W’, ’AH’, ’K’, ’T’, ’ER’, ’F’, ’AH’, ’K’)	(’AH’, ’K’, ’T’), (’IY’, ’F’), (’K’, ’UH’), (’W’, ’AA’, ’K’, ’T’), (’W’, ’AH’, ’K’, ’T’, ’ER’, ’F’, ’AH’, ’K’)	AND IF KNEW WANT WONDERFUL	0.04	AND IF YOU WANT WONDERFUL	0.05
FOR A BRIEF TIME	(’F’, ’AO’, ’W’), (’AH’), (’P’, ’W’, ’IY’, ’F’), (’T’, ’AH’, ’P’)	(’AO’, ’AO’, ’W’), (’AH’), (’P’, ’W’, ’IY’, ’F’), (’T’, ’AH’, ’P’)	FOR I THIS TYPE	0.04	FOR A BIG TYPE	0.05
IT WILL CHANGE LIVES	(’IY’, ’T’), (’W’, ’IY’, ’K’), (’CH’, ’EY’, ’K’, ’CH’), (’K’, ’IY’, ’F’, ’T’)	(’T’, ’T’), (’W’, ’IY’, ’K’), (’CH’, ’EY’, ’K’, ’CH’), (’K’, ’IY’, ’F’, ’T’)	THIS WILL CHANGE LIVES	0.04	THIS WILL CHANGE LIVES	0.05
I THINK IT’S BRILLIANT	(’AH’), (’T’, ’IY’, ’K’, ’K’), (’IY’, ’T’, ’T’), (’P’, ’W’, ’IY’, ’K’, ’K’, ’AH’, ’K’, ’T’)	(’AH’), (’T’, ’IY’, ’K’), (’IY’, ’T’, ’T’), (’P’, ’W’, ’IY’, ’K’, ’K’, ’AH’, ’K’, ’T’)	I THINK IT’S BRILLIANT	0.04	I THINK IT’S BRILLIANT	0.04
BUT IT’S A DECENT SIZE	(’P’, ’AH’, ’T’), (’IY’, ’T’, ’T’), (’AH’), (’T’, ’IY’, ’T’, ’AH’, ’K’, ’T’), (’T’, ’AH’, ’T’)	(’P’, ’AH’, ’T’), (’IY’, ’T’, ’T’), (’AH’), (’T’, ’IY’, ’T’, ’AH’, ’K’, ’T’), (’T’, ’AH’, ’T’)	BUT IT’S A DECENT SUSS	0.05	BUT IT’S A DECENT SIZE	0.05

## Data Availability

The data utilised in this research comes from the audiovisual datasets BBC-LRS2 and LRS3-TED that were compiled by the Visual Geometry Group at the University of Oxford.
